# Cost-effectiveness analysis of a community-based colorectal cancer screening program in Shanghai, China

**DOI:** 10.3389/fpubh.2022.986728

**Published:** 2022-10-07

**Authors:** Hongli Jiang, Peng Zhang, Kai Gu, Yangming Gong, Peng Peng, Yan Shi, Dashan Ai, Wen Chen, Chen Fu

**Affiliations:** ^1^School of Public Health, Fudan University, Shanghai, China; ^2^Shanghai Municipal Center for Disease Control and Prevention (SCDC), Shanghai, China; ^3^Department of Radiation Oncology, Shanghai Cancer Center, Fudan University, Shanghai, China; ^4^Shanghai Clinical Research Center for Aging and Medicine, Shanghai, China

**Keywords:** cost-effectiveness, colorectal cancer, mass screening, community screening, China

## Abstract

**Background:**

Since 2011, through the Community-Based Colorectal Cancer Screening Program in Shanghai, China (SHcsp), residents aged >50 years were offered initial colorectal cancer screening using the fecal immunochemical test (FIT) and risk assessment questionnaire (RAQ) for free. Colonoscopy was then recommended for positive results.

**Objective:**

To evaluate the cost-effectiveness of the Community-Based Colorectal Cancer Screening Program in Shanghai, China from the payer perspective.

**Methods:**

This analysis estimated the long-term cost and effectiveness of the 2014–2016 SHcsp based on real-world follow-up data from the SHcsp database, Shanghai Cancer Registry System, vital statistics from Shanghai Municipal Center for Disease Control and Prevention and inpatient CRC expense data from hospitals. A decision-tree model and Markov model were constructed to simulate the 25-year health outcomes. The screening branch was the cohort with a definite diagnosis of adenoma, advanced adenoma, and CRC. The other branch was residents who were neither screened nor treated until CRC symptoms appeared. A payer prospective was adopted to measure direct costs and effectiveness by life years (LYs) and quality-adjusted life years (QALYs) gained, and were discounted by 3%. Stimulation robustness was tested by one-way sensitivity analysis.

**Results:**

Of 1,097,656 residents, 13,250 were diagnosed with adenoma, advanced adenoma, or CRC. Assuming those had not been found through screening, SHcsp resulted in 1,570.1 LYs and 13,984.3 QALYs gained at an extra cost of USD9.96 million. The incremental cost-effectiveness ratio (ICER) was USD6,342.02 per LY and USD712.08 per QALY obtained, far below the threshold of USD59,598 of three-time GDP per capita in Shanghai.

**Conclusion:**

The SHcsp was cost-effective than no screening strategy. The results were generalisable to the Chinese population for mass CRC screening.

## Introduction

Colorectal cancer (CRC) is the fourth most common cancer in China; 4.29 million new cases were diagnosed in 2018, accounting for 1/4 of new cases worldwide. It is also the second most common cause of cancer-related deaths in China, with 2.81 million CRC-related deaths in 2018, with a rapid growth rate (1). Accumulated evidence from global CRC screening programs strongly suggests that CRC screening increases the proportion of early detection and reduces CRC incidence and mortality (2–5). Epidemiological studies have shown that annual mortality of CRC in the United States fell by 3.9% every year from 2002 to 2009, and 53% of the reduction was attributed to screening (6). Due to the lack of CRC screening in China, only 12% of the newly diagnosed cases per year were in the early stages in 2012 (7), compared with 39% of US cases in the same period (8).

Colorectal cancer screening programs have been conducted worldwide. Over half of European countries, such as Germany, France, the United Kingdom, Denmark, and the Netherlands, have implemented CRC screening programs, and several countries have established the guidelines for CRC screening (9–13). These programs mostly targeted the natural population over 50 years old instead of the high-risk population, used the fecal immunochemical test (FIT) as initial screening and colonoscopy as diagnostic tests, and operated regularly and periodically. South Korea, Croatia, and some developing countries have also explored the feasibility of CRC screening (14, 15). In the 1970s, some small cities in China carried out a few pilot screening programs for CRC among local residents, such as Haining and Jiashan in Zhejiang Province (16, 17). Recently, metropolitan areas in mainland China, including Shanghai, Tianjin, and Guangzhou, have promoted screening programs for CRC on a larger scale (18–20). In addition, health economics research has shown CRC screening programs to be cost-effective in France, England, and Belgium (4, 9, 21, 22). However, the long-term outcomes and economic evidence for mass CRC screening programs are unknown, apart from cohort simulations in China and other developing countries (23).

As one of the most developed cities in China, Shanghai faces the threat of a dramatically increased incidence and mortality of CRC, which are second and fourth highest among all cancers, respectively (24). As an aging megalopolis in China, Shanghai is the pioneer of mass CRC screening for community residents with the most extensive participation. After a pilot CRC screening program in the Qibao community, the Community-Based Colorectal Cancer Screening Program in Shanghai (SHcsp) was officially launched in 2011 to provide free colorectal cancer screening for community residents over 50 years old every 3 years (18). Approximately 4 million community residents were estimated to be eligible, and ~1 million were planned to be included every 3 years. During 2011–2013, SHcsp made significant achievements (18), and during 2014–2016 provided 1,097,656 initial screenings.

**Figure 1:**
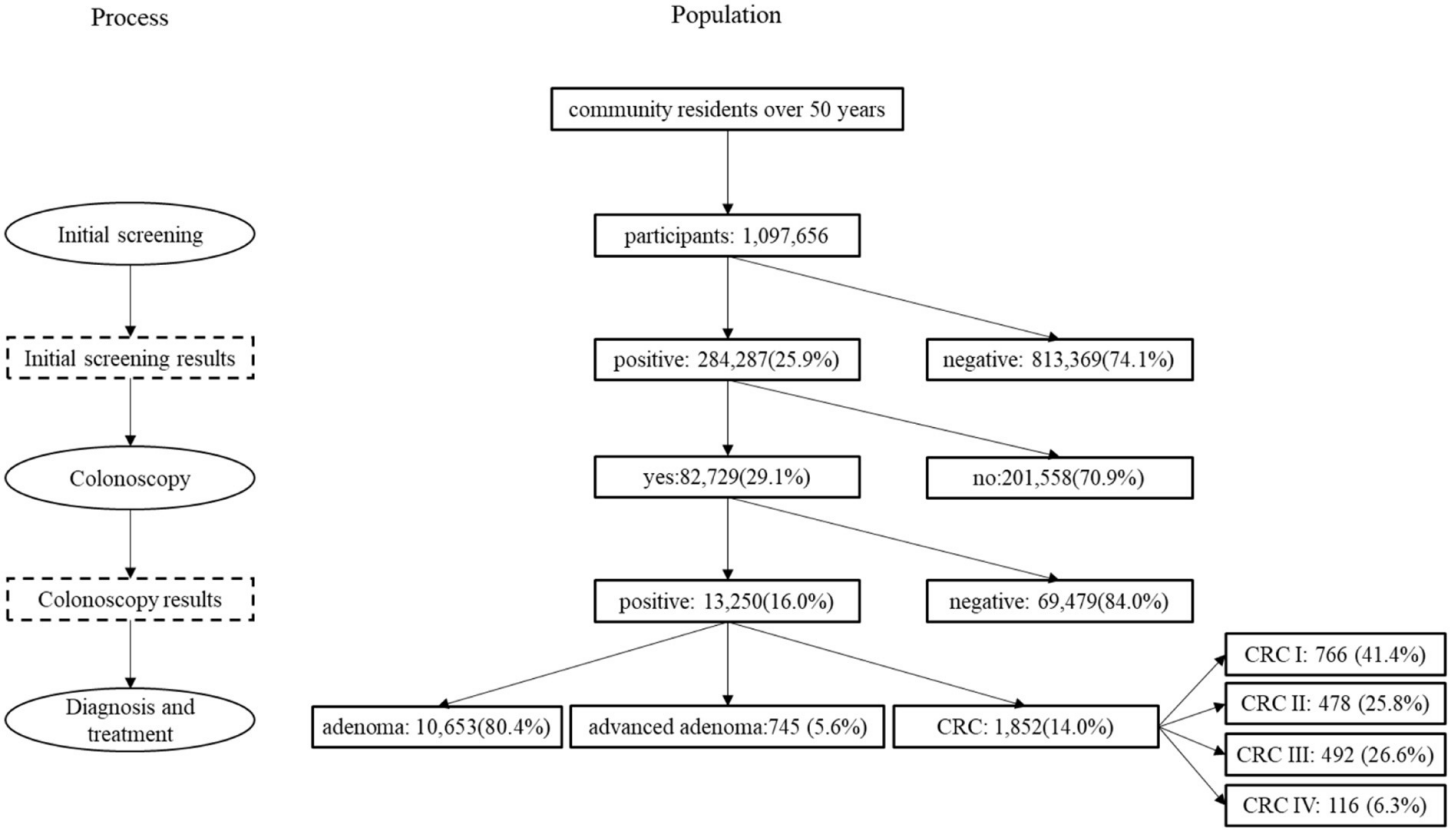
Process of 2014–2016 SHcsp.

The entire process can be divided into four steps (shown in Figure 1): (1) Sponsored by the local government and organized by the community health centers, the initial screening was free for residents after registration and completion of FIT and risk assessment questionnaire (RAQ). (2) Community physicians informed participants of their initial screening results and recorded them in the SHcsp database. (3) Participants with positive screening results based on either FIT or RAQ results were suggested by doctors in community health centers to undergo colonoscopy in hospitals, which was covered by the local basic health insurance schemes. As a diagnostic test, colonoscopy results confirmed the patients' health status. (4) Patients diagnosed with adenoma, advanced adenoma, or CRC were treated differently. Almost all adenomas and advanced adenomas were surgically removed during colonoscopy in outpatient settings. Only a few advanced adenoma and CRC patients required hospitalization for further treatment, and CRC patients were reported to the Shanghai Cancer Registry System (SCRS). Patients were followed up by doctors in community health centers for one year after their initial screenings, which were updated in the SHcsp database.

SHcsp is rare in community-based populations and extensive participation. It is essential to evaluate not only the short-term results of the implementation, but also the long-term health effectiveness (18). Other CRC screening programs in China were limited to evaluate the health value of community-based CRC screening programs because of the small sample size, short-term implementation, or lack of detailed follow-up data on diagnosis, treatment, and survival results. The cohort of one million people, strict procedures, and accessible data of SHcsp are suitable for analyzing the cost-effectiveness of mass CRC screening programs and providing real-world economic evidence in China.

This study, based on real-world data collected from the SHcsp database, Shanghai Cancer Registry System managed by Shanghai Municipal Center for Disease Control and Prevention (SCDC), vital statistics from SCDC and inpatient information from hospitals, constructed a decision tree model and a Markov model to stimulate the cost and effectiveness over 25 years from the payer perspective. The incremental cost-effectiveness ratio (ICER) was reported to show the results, and a sensitivity analysis was performed to confirm the validity of this study.

## Materials and methods

### Model overview

Of 1,097,656 SHcsp participants, positive initial screening results were detected in 284,287 and 82,729 of them underwent colonoscopy. Finally, 13,250 patients were diagnosed: 10,653 had adenoma, 745 had advanced adenoma, and 1,852 were CRC patients without obvious symptoms. Because SHcsp prevented disease progression in the 13,250 patients who were finally diagnosed and treated, our study focused on the long-term effectiveness of these patients. Their improved health condition directly and accurately reflected the real screening performance, rather than simulating a hypothetical cohort based on CRC prevalence rate, sensitivity and specificity of FIT and RAQ, detection rates, and adherence to colonoscopy (shown in Figure 1).

The target population can be divided into two groups: the screening group, with 13,250 residents diagnosed positive; and the comparison group, as counterfactual reference, residents who did not participate in the screening and whose diseases progressed naturally until their clinical symptoms of CRC emerged and they sought medical care.

The essential difference between the two groups was that in the screening group, adenoma, advanced adenoma, and more early stage CRC patients were diagnosed and treated, which greatly prevented or slowed down the disease progression. However, in the comparison group of natural disease processes, these patients could hardly be detected because they were normally asymptomatic.

### Model structure

To represent the screening results and further progress of CRC, a model combining a decision tree and a Markov model was constructed (9, 21, 22, 25, 26). The screening program only changed the initial health state distribution of residents shown by the decision tree, but did not influence the further transition probabilities between health states in the Markov model. The Treeage Pro 2018 was used to generate the cost-effectiveness estimates.

**Figure 2:**
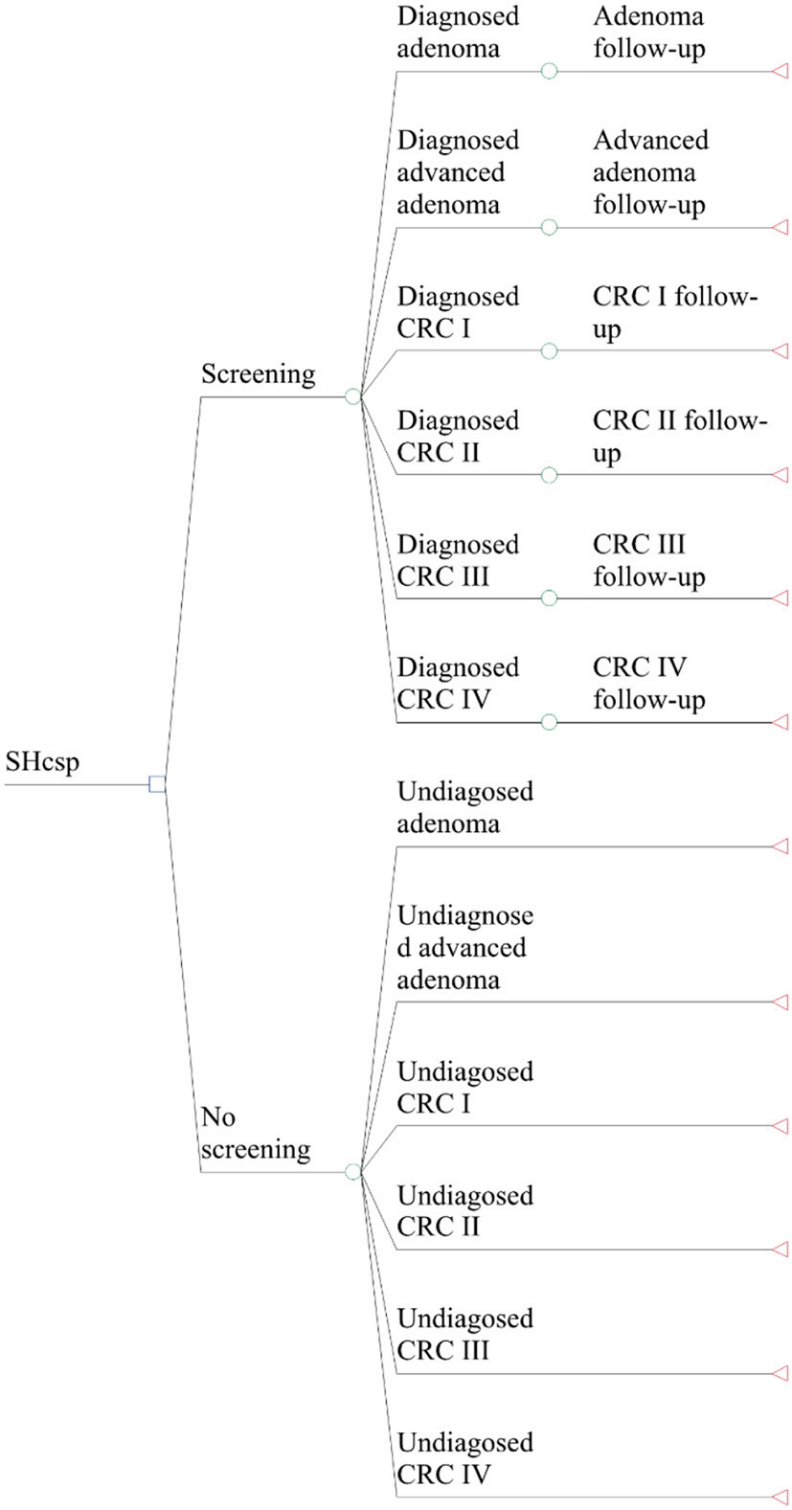
Structure of decision tree model.

According to the Tumor-Node-Metastasis (TNM) stages recommended by American Joint Committee on Cancer (AJCC) (27), a decision tree was constructed to determine the initial distribution in the Markov model (21). Based on the diagnostic results of colonoscopy for 13,250 residents, the initial distribution of the screening group and the comparison group was divided into 12 initial states (shown in Figure 2).

**Figure 3:**
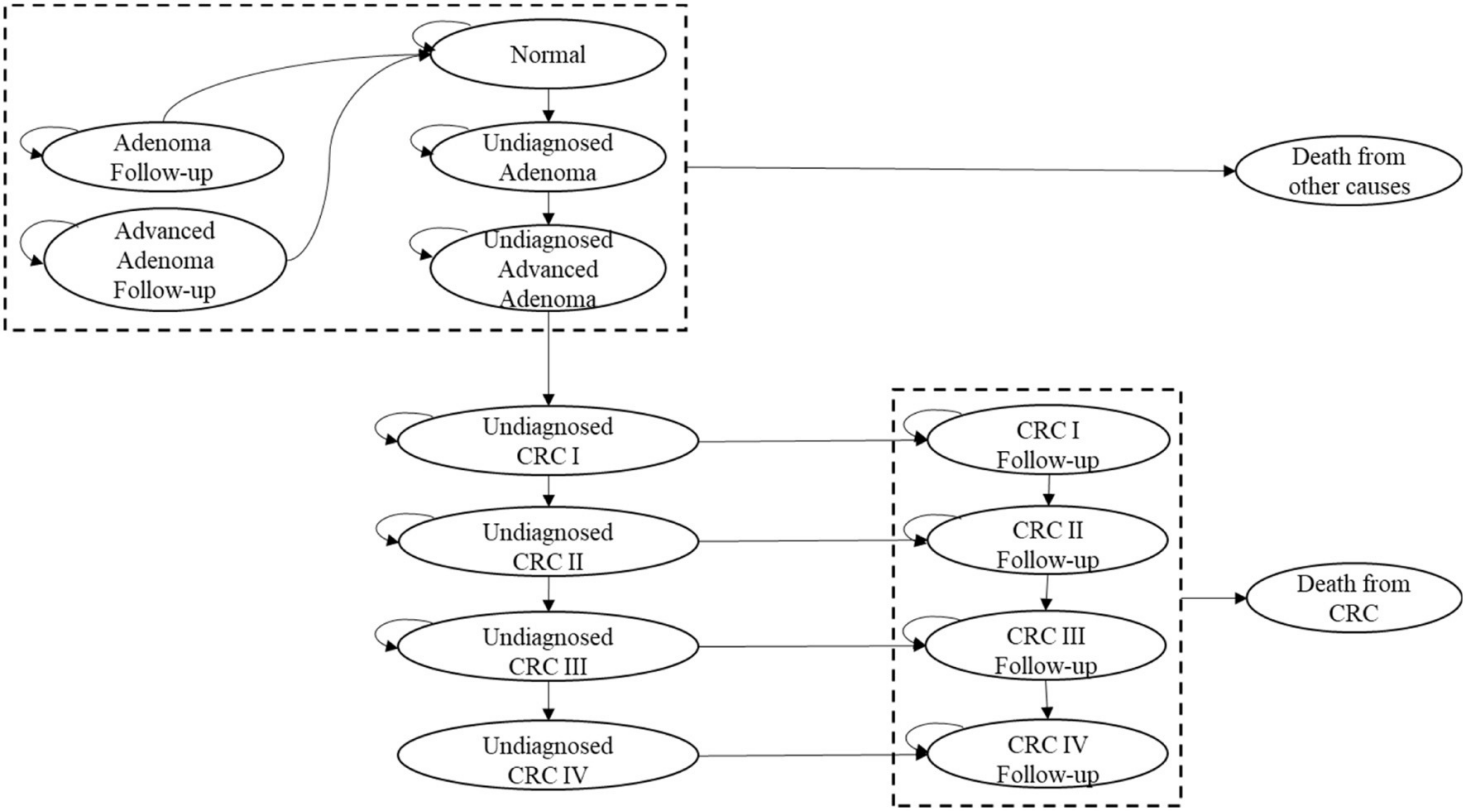
Structure of the Markov model.

We classified the 15 possible health states of the two groups in the Markov model (shown in Figure 3): normal (no adenomas or cancer), adenoma (low-risk adenoma with a diameter <10 mm), advanced adenoma (high-risk adenoma with a diameter bigger than 10 mm), adenoma follow-up (5 years after adenoma polypectomy), advanced adenoma follow-up (5 years after advanced adenoma polypectomy), undiagnosed CRC Stage I, Stage II, Stage III, and Stage IV; CRC follow-up Stage I, Stage II, Stage III, and Stage IV; death from CRC, and death from other causes. Transitions occur once in each annual cycle over 25 years, considering the starting age for screening and life expectancy of Shanghai residents (25, 26).

The assumptions to simplify our model are as follows:

(1) During 2014–2016, only unscreened natural residents were included in the SHcsp. Neither repeat screening nor complications of initial screening were considered, due to the low incidence.
(2) In the screening group, residents diagnosed with adenoma or advanced adenoma underwent immediate polypectomy during colonoscopy and returned to the normal state after a 5-year follow-up. In the comparison group, adenoma or advanced adenoma patients could not be detected or treated, and no death occurred.
(3) In the screening group, patients diagnosed with CRC were assumed to undergo radical surgery and transition to the corresponding follow-up states with no recurrence. In the comparison group, only a small number of undiagnosed patients were diagnosed and treated due to clinical symptoms and timely consultations.

### Model parameters

The model was built as much as possible with real-world follow-up data from the SHcsp database, Shanghai Cancer Registry System, and inpatient CRC expense data from hospitals, supplemented by the literature where necessary. The half-cycle correction was applied, and the annual discount rate was assumed to be 3% (2, 3, 23, 28, 29). The parameters of the model are listed in Table 1.

**Table 1:** Parameters of the Markov model for SHcsp.

**Parameters**	**Base-case**	**Reference**
**Distribution of screening group**		
Adenoma follow-up	0.8040	SHcsp follow-up data
Advanced adenoma follow-up	0.0562	SHcsp follow-up data
CRC I follow-up	0.0578	SHcsp follow-up data
CRC II follow-up	0.0361	SHcsp follow-up data
CRC III follow-up	0.0371	SHcsp follow-up data
CRC IV follow-up	0.0088	SHcsp follow-up data
**Distribution of assumed no-screening group**		
Adenoma	0.8040	SHcsp follow-up data
Advanced adenoma	0.0562	SHcsp follow-up data
Undiagnosed CRC I	0.0578	SHcsp follow-up data
Undiagnosed CRC II	0.0361	SHcsp follow-up data
Undiagnosed CRC III	0.0371	SHcsp follow-up data
Undiagnosed CRC IV	0.0088	SHcsp follow-up data
**Transition probability (per year)**		
From normal to adenoma	0.0160	(30)
From adenoma to advanced adenoma	0.0200	(30)
From advanced adenoma to undiagnosed CRC I	0.0326	(31)
From undiagnosed CRC I to undiagnosed CRC II	0.2400	(32, 33)
From undiagnosed CRC I to CRC I follow-up	0.2000	(32)
From undiagnosed CRC II to undiagnosed CRC III	0.3600	(32, 34)
From undiagnosed CRC II to CRC II follow-up	0.2000	(32)
From undiagnosed CRC III to undiagnosed CRC IV	0.1750	(32, 34)
From undiagnosed CRC III to CRC III follow-up	0.6500	(32)
From undiagnosed CRC IV to CRC IV follow-up	1.0000	(32)
Death from other causes	0.0175	(35)
Death from CRC I follow-up	0.0218	Vital statistics and SHcsp follow-up data
Death from CRC II follow-up	0.0458	Vital statistics and SHcsp follow-up data
Death from CRC III follow-up	0.0994	Vital statistics and SHcsp follow-up data
Death from CRC IV follow-up	0.2028	Vital statistics and SHcsp follow-up data
Costs ($ per capita)^*^		
Cost for initial screening and colonoscopy	1,271.90	Questionnaire and official prices data
Treatments for advanced adenoma	1,108.82	Inpatient expense data
Treatments for CRC I	8,566.63	Inpatient expense data
Treatments for CRC II	10,554.70	Inpatient expense data
Treatments for CRC III	13,395.73	Inpatient expense data
Treatments for CRC IV	11,177.51	Inpatient expense data
**Utility**		
Normal	1.000	Assumption
Adenoma	0.871	(25)
Advanced adenoma	0.827	
Stage I CRC	0.829	
Stage II CRC	0.860	
Stage III CRC	0.814	
Stage IV CRC	0.738	
**Discount rate**		
Discount rate of costs	3%	(2, 3, 23, 28, 29)
Discount rate of life years gained	3%	(2, 3, 23, 28, 29)

* All costs converted into dollars using 2014–2016 mean exchange rate of USD $1 = RMB ¥ 6.3743.

#### Clinical data

The initial distribution was calculated based on the follow-up results from the SHcsp database. The 13,250 diagnosed patients were divided into six states by the decision tree: adenoma follow-up, advanced adenoma follow-up, Stage I CRC follow-up, Stage II CRC follow-up, Stage III CRC follow-up, and Stage IV CRC follow-up. These patients were treated immediately after diagnosis and returned to the corresponding follow-up status.

The crude mortality of residents was reported by vital statistics from SCDC. The CRC mortalities of the four CRC stages were calculated, respectively, as 5-year survival rates derived from the SCRS in the same period. The 5-year survival rates of the four stages were converted to annual survival rates. The CRC mortality rates of the four CRC stages were then determined and are shown in Table 2 using the following formula: Other transition probabilities between underlying disease states were mainly based on previously validated studies.


$$\begin{array}{l} \;{SurvivalRate}_{annually}\;=\;{{SurvivalRate}_{5-year}}^{\frac{1}{5}}\\ \;Mortality\;=\;{1-SurvivalRate}_{annually} \end{array}$$

**Table 2:** Survival rates and mortality of the four CRC stages.

	**5-year survival rate**	**Annual survival rate**	**Mortality**
CRC I	0.8957	0.9782	0.0218
CRC II	0.7912	0.9542	0.0458
CRC III	0.5924	0.9006	0.0994
CRC IV	0.3220	0.7972	0.2028

#### Costs

From the payer perspective, direct costs of SHcsp during 2014–2016 were collected, including initial screening costs, colonoscopy costs, and treatment costs for advanced adenoma and CRC patients. There were different numbers of participants at each step of the whole screening process and treatments afterwards, as shown in Figure 1 and Table 3. The mean exchange rate of US dollar to Renminbi during 2014 to 2016 was used, where USD1 equals RMB6.3743.

**Table 3:** Costs for SHcsp and input costs in the Markov model.

	***N***	**Total costs ($)**	**Per capita cost included in the model ($)**
Costs for initial screening and colonoscopy allocated to the diagnosed patients	13,250	16,852,628	1,271.90
Initial screening	1,097,656	3,721,054	280.83
Colonoscopy	82,729	13,131,574	991.06
Treatments for advanced adenoma	745	826,071	1,108.82
Treatments for CRC I	766	4,814,446	8,566.63
Treatments for CRC II	478	3,704,700	10,554.7
Treatments for CRC III	492	4,835,859	13,395.73
Treatments for CRC IV	116	950,088	11,177.51

All costs converted into dollars using 2014–2016 mean exchange rate of USD $1 = RMB ¥ 6.3743.

During 2014–2016, initial screening costs for 1,097,656 participants included costs of purchasing materials, organizing programs, publicizing, communicating with residents, and manpower inputs. The costs of materials, organization, and publicity, which were sponsored by the municipal and district governments, were the actual expenses of SHcsp and were collected from organizers such as community health centers and SCDC. To calculate other costs, our staff designed a questionnaire and interviewed health care providers and administrators from community health service centers and SCDC, who implemented SHcsp in practice. We collected information on staff man-hours spent on SHcsp and their monthly salaries. The total cost for initial screening was USD16,852,628.

Colonoscopy costs for 82,729 participants were calculated by multiplying the number of patients with the costs per patient. According to the guidelines and clinical practice in Shanghai, the average costs included the costs of colonoscopy and preoperative tests, including chest radiography, electrocardiogram, and liver function tests. The flat fees for these tests were summed up based on the official prices in Shanghai. The cost for colonoscopy was USD158.73 per capita.

As mentioned before, 13,250 residents who were detected were entered in the model, so that the costs of initial screening and colonoscopy were converted into average costs of screening for every 13,250 residents as model inputs. The input cost for initial screening and colonoscopy was USD1,271.90 in total.

The treatment costs for 10,653 adenoma patients were included in the colonoscopy costs. The vast majority of adenoma polypectomies were completed during the colonoscopy procedure in an outpatient setting with limited extra cost, as suggested by health care providers.

Treatment costs for 745 advanced adenoma and 1,852 CRC patients were calculated by multiplying the number of patients with the median treatment costs for each status. Patients' health expenses were collected from local hospitals by matching the unique IDs of registered residents in SHcsp. The treatment costs were defined as the total hospitalization expenses of each diagnosed patient who underwent operations and other treatments in hospitals within 1 year after screening. After data cleansing, we deleted outliers and used median treatment costs due to the partial distribution. The median treatment costs for advanced adenoma and four stages of CRC were USD1,109, USD8,567, USD10,555, USD13,396, and USD11,178 per patient, respectively.

#### Effectiveness

As for effectiveness in our analysis, health outcomes were valued in terms of life years (LYs) gained as the main results, and utilities of possible health states of Chinese residents and patients were obtained from the literature. We estimated cumulative life years and quality-adjusted life years over 25 years using the model to reflect the survival condition of the population.

As recommended by the World Health Organization (36), three times the local gross domestic product (GDP) per capita was used as the threshold to estimate whether SHcsp was cost-effective. In 2017, the GDP per capita in Shanghai was USD19,866 per capita.

### Uncertainty

**Figure 4:**
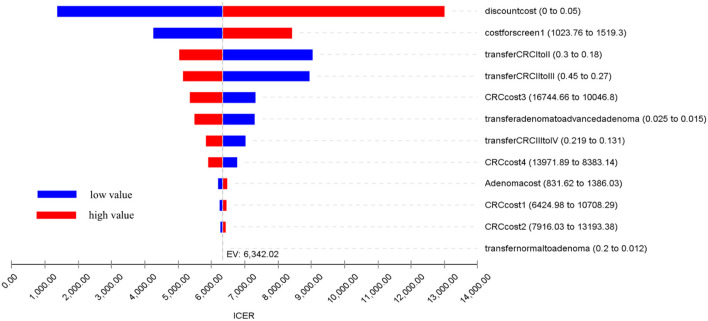
Tornado diagram of the results of the one-way sensitivity analysis.

A one-way sensitivity analysis was performed on the model parameters: (1) Transition probabilities between health states: the transition probabilities from normal to adenoma, from adenoma to advanced adenoma, and between different CRC stages. (2) The costs for diagnosis and treatment: the costs of colonoscopy and treatment costs for the four CRC stages. (3) Discount rates. These explored the uncertainty of the model results due to important parameters, especially those not derived from program data. The substituted parameters used in the analysis were independently varied by ±25% from the base-case value. The results of the sensitivity analysis are reported in Table 5 and presented in a tornado diagram in Figure 4. A cost-effectiveness threshold was also used.

## Results

### Incremental cost-effectiveness

The estimates of cost and effectiveness of SHcsp are shown in Table 4. The total costs of the screening group and the no screening group were USD37.17 million and USD27.21 million respectively. The incremental costs for SHcsp were USD9.96 million.

**Table 4:** Comparison of the cost effectiveness between SHcsp and no screening.

	**SHcsp screening**	**No screening**
**Effectiveness**		
Deaths from CRC	1,226	1,821
Deaths from other causes	4,067	3,831
Life years gained (LYs)	187,377.70	185,807.40
Quality-adjusted life years (QALYs)	174,587.20	160,602.90
Incremental life years (LYs)	1,570.1.10	–
Incremental quality-adjusted life years (QALYs)	13,984.30	–
**Costs (million $)**		
Total costs	37.17	27.21
Incremental costs	9.96	–
**Incremental cost-effectiveness ratio**		
Incremental costs/LY gained ($/LY)	6,342.02	–
Incremental costs/QALY gained ($/QALY)	712.08	–

After 25 years, a total of 187,377.7 LYs were cumulated in the screening group and 185,807.4 LYs were cumulated in the no screening group. Compared with no screening strategy, the SHcsp gained additional effectiveness of 1,570.1 LYs with extra costs of USD9.96 million. The ICER was $6,342.02 per LY gained.

Considering the health utility of patients, we also estimated the cost-utility of SHcsp. After 25 years, a total of 174,587.2 QALYs were cumulated in the screening group and 160,602.9 QALYs were cumulated in the no screening group. Compared with no screening, additional 13,984.3 QALYs were gained with same extra costs of USD9.96 million. Only USD712.08 was required for each QALY gained.

The Shanghai GDP in 2017 was US$ 19,866 per capita. The ICER of SHcsp was under Shanghai GDP per capita in 2017 and far below the threshold of three times the Shanghai GDP per capita (USD59,598). These results prove that SHcsp is highly cost-effective.

### Sensitivity analysis

A one-way sensitivity analysis was performed on the relevant model parameters, as listed in Table 5. A tornado diagram graphically displays the results of one-way sensitivity analysis of the 14 parameters with their respective impacts on ICER (Figure 2).

**Table 5:** One-way analysis of the parameters in the Markov model.

**Model parameters**	**Base-case**	**Sensitivity analysis**		**ICER($/LY)**	
		**Low value**	**High value**	**Low value**	**High value**
Discount rate	3%	0%	5%	1,360.84	13,013.86
**Costs for initial screening and colonoscopy per diagnosed patient**					
Colonoscopy per diagnosed patient	158.73	119.05	198.41	4,251.19	8,432.84
**Costs for treatments**					
Advanced adenoma	1,108.82	831.62	1,386.03	6,210.49	6,473.55
CRC I	8,566.63	6,424.98	10,708.29	6,235.01	6,449.02
CRC II	10,554.70	7,916.03	13,193.38	6,262.05	6,421.98
CRC III	13,395.73	10,046.80	16,744.66	7,324.67	5,359.37
CRC IV	11,177.51	8,383.14	13,971.89	6,774.84	5,909.19
**Transition probabilities (from – to)**					
Normal-adenoma	0.016	0.012	0.020	6,342.02	6,342.02
Adenoma-advanced adenoma	0.02	0.015	0.025	7,306.69	5,478.46
Undiagnosed CRC I-undiagnosed CRC II	0.24	0.180	0.300	9,003.41	5,039.56
Undiagnosed CRC II-undiagnosed CRC III	0.36	0.270	0.450	8,958.52	5,145.85
Undiagnosed CRC III-undiagnosed CRC IV	0.175	0.131	0.219	7,025.71	5,837.66

The one-way analysis demonstrates the robustness of the results of the cost-effectiveness analysis, indicating that our model was relatively insensitive to changes in parameter values. The most sensitive of the parameters was the discount rate, with a range of 0–5% leading to a change in ICER from USD1,360.84 to USD4,137.28 per LY gained. The costs for screening and treatment had relatively limited impacts on cost-effectiveness, such as the annual transition probabilities between health states. All the results were still under the threshold.

## Discussion

This study demonstrates the notable cost-effectiveness of mass CRC screening for Chinese community residents. SHcsp brought benefits for 13,250 residents with more precancerous lesions, causing a higher detection rate of early stage CRC and providing the chance to prevent and treat CRC, which directly relieved the burden of disease for patients and society. In the long term, after the 25-year simulation, there was more health improvement and life years gained. The incremental cost-effectiveness ratio was USD6,342.02 per LY and USD712.08 per QALY gained, which was far below the threshold of USD59,598. The cost-effectiveness results of the base-case scenario were shown to be robust by sensitivity analysis.

China has only implemented CRC screening programs in a few cities. However, no economic evaluation of mass CRC screening has been performed. There are also limited studies that have evaluated completed programs globally (4, 9, 21). On the contrary, most of the economic evaluations of CRC screening have been based on simulative cohorts using parameters from the literature that are not appropriate for specific populations in other countries (3, 5, 23, 25, 26, 29, 31). Seizing the opportunity to analyse a long-term, large-scale, and well-finished program, our study aimed to analyse the cost-effectiveness of mass CRC screening programs in developing countries. Another advantage was the SHcsp data accessibility and integrity of other population health indicators in Shanghai, leading to more convincing results. Our study provides new evidence that mass CRC screening has effective outcomes and economic influence in China.

Although there were false-positive results in practice, these residents' health conditions were not influenced by SHcsp, but all screening and colonoscopy expenses were included in the analysis. To directly and accurately reflect real-world effectiveness, our study focused on 13,250 diagnosed and treated patients. For the same reason, we did not make ideal model assumptions regarding such factors as the disease morbidity rate, colonoscopy compliance of patients with positive initial screening (2), or sensitivity and specificity of the screening tests (29), thereby reducing bias and distortion. Our model combined a decision tree, indicating the main results of the screening, with a Markov model of long-term health state transitions. The Markov model was established through careful consideration, and 15 health states might be closer to the natural disease progression of CRC (2, 3, 5, 23).

The transition probabilities between health states were calculated according to the actual incidence and mortality rates in Shanghai. These local and detailed information included data from the SHcsp follow-up database, the Shanghai Cancer Registry System of SCDC, vital statistics from SCDC, and clinical records from hospitals. The integrity of multiple local data resources, the initial distribution, and costs were more reliable and suitable than previous studies, which mainly used simulative cohorts and data from other original studies (3, 5, 21, 23, 25, 26, 29, 31).

This study has several policy implications. Mass CRC screening programs are feasible, effective, and cost-effective for the Chinese population and in the current situation. The initial screening strategy combining FIT with RAQ proved to be scientific and suitable (18). These results should be generalisable to the Chinese population, because the clinical and cost parameters were mostly based on real-world data from the completed program (3, 25). Policymakers and healthcare providers in other regions should also use care when taking this local evidence into consideration (26, 29).

This study has a few limitations. First, like all model simulations, our model structures were simplified from the natural history of CRC. All treatments were regarded as radical treatment, so recurrences after treatment were not considered (21, 26, 31). Second, due to the limitations of available data, the probabilities of cancer progression were assumed to be equivalent in different ages and between the two groups, and the medians of treatment costs were used due to loss to follow-up of every treatment, leaving room for improvement (22). Last but not least, as other related studies have mentioned, it is difficult to determine transition probabilities without the basis of solid epidemic studies or pathological examination of colorectal adenoma and cancer, so some of the transition probabilities were derived from estimates in the literature (21, 31). For further research, basic epidemic studies, accurate follow-ups and registered data are required. In addition to direct costs, indirect costs should be measured to shed light on the comprehensive burden of CRC (5, 23, 25, 29), and investigations of patients' life condition and health-related utility should be conducted.

In conclusion, using real-world data from the completed program and a valid combined model, this study demonstrated that mass CRC screening, SHcsp during 2014–2016, was cost-effective compared to no screening, improving the general health state and relieving the disease burden. Mass CRC screening with a suitable strategy and optimized process is feasible, beneficial, and economical.

## Data availability statement

The original contributions generated for the study are included in the article/supplementary material, further inquiries can be directed to the corresponding author/s.

## Author contributions

HJ and PZ contributed to the conception and design of the study under the supervision of WC and CF. KG, YG, and YS performed the data collection. PZ, KG, and DA conducted statistical analysis. PZ wrote the manuscript and revised as needed under the guidance of HJ and WC. WC and CF supervised the overall study and provided feedback throughout all stages. All authors contributed to the article and approved the submitted version.

## Funding

Research support funding for this project was received from Shanghai Science and Technology Achievement Transformation and Industrialization Project (18401933403). This research is partially supported by Shanghai Clinical Research Center for Aging and Medicine (19MC1910500).

## Conflict of interest

The authors declare that the research was conducted in the absence of any commercial or financial relationships that could be construed as a potential conflict of interest.

## Publisher's note

All claims expressed in this article are solely those of the authors and do not necessarily represent those of their affiliated organizations, or those of the publisher, the editors and the reviewers. Any product that may be evaluated in this article, or claim that may be made by its manufacturer, is not guaranteed or endorsed by the publisher.
